# Dried Urine Microsampling Coupled to Liquid Chromatography—Tandem Mass Spectrometry (LC–MS/MS) for the Analysis of Unconjugated Anabolic Androgenic Steroids

**DOI:** 10.3390/molecules25143210

**Published:** 2020-07-14

**Authors:** Michele Protti, Camilla Marasca, Marco Cirrincione, Angelo E. Sberna, Roberto Mandrioli, Laura Mercolini

**Affiliations:** 1 Research Group of Pharmaco-Toxicological Analysis (PTA Lab), Department of Pharmacy and Biotechnology (FaBiT), Alma Mater Studiorum-University of Bologna, 40126 Bologna, Italy; michele.protti2@unibo.it (M.P.); camilla.marasca2@unibo.it (C.M.); marco.cirrincione2@unibo.it (M.C.); 2 Sport Medicine, Enna Local Health Unit, 94100 Enna, Italy; angsber@gmail.com; 3 Department for Life Quality Studies, Alma Mater Studiorum-University of Bologna, 47921 Rimini, Italy; roberto.mandrioli@unibo.it

**Keywords:** anabolic androgenic steroids, anti-doping, dried urine spots (DUS), LC-MS/MS, microsampling, neuroprotection, volumetric absorptive microsampling (VAMS)

## Abstract

Testing and monitoring anabolic androgenic steroids in biological fluids is a key activity in anti-doping practices. In this study, a novel approach is proposed, based on dried urine microsampling through two different workflows: dried urine spots (DUS) and volumetric absorptive microsampling (VAMS). Both techniques can overcome some common drawbacks of urine sampling, such as analyte instability and storage and transportation problems. Using an original, validated liquid chromatography–tandem mass spectrometry (LC-MS/MS) method, exogenous and endogenous unconjugated steroids were analysed. Despite the limitations of microsampling volume, good sensitivity was obtained (limit of quantitation ≤1.5 ng/mL for all analytes), with satisfactory precision (relative standard deviation <7.6%) and absolute recovery (>70.3%). Both microsampling platforms provide reliable results, in good agreement with those obtained from urine.

## 1. Introduction

Recent decades have witnessed the worrying phenomenon of a disproportionate increase in the use of performance-enhancing substances, in particular anabolic androgenic steroids (AAS), not only among high-level professional athletes, but also among all other athletes up to amateur ones [1]. This is mainly related to the prompt availability of powerful AAS for the average user: in fact, due to the proliferation of online retail stores, these substances can be purchased on the web and shipped directly home through the postal service [2]. The phenomenon has spread mostly in the framework of gym circuits, where athletes abuse AAS in order to increase muscle strength and size, and the lean-to-fat body mass ratio, thus amplifying the physical exercise outcomes [3]. These results, however, also entail serious side effects: endocrine effects due to competition with cortisol, virilisation, acne, gynecomastia and testicular atrophy, increase of cardiovascular issues, liver complications [4,5]. It has also been proven that individuals abusing AAS incur more easily in neuropsychiatric disorders such as anxiety, paranoia and depression [6]. Although the possible role of AAS in causing either neurotoxicity or neuroprotection is still unclear, evidence of prevalent neurotoxic effects after administration of synthetic AAS is mounting [7,8,9]. As a consequence, AAS use is strictly prohibited by the World Anti-Doping Agency (WADA) [10], both in-competition and out-of-competition, and for this reason it is of the utmost importance to develop and update analytical tools to reliably detect their use and possible abuse. These tools could also be useful to study and clarify the activity of AAS administration on neuronal health and the effectiveness of prospective neuroprotective agents. In the recent past, gas chromatography (GC) coupled to mass spectrometry (MS) has been routinely applied to human urine for the screening and confirming analysis of AAS and metabolites [11,12,13,14]. However, AAS analysis by GC-MS requires time consuming derivatisation steps and still suffers from sensitivity and selectivity issues for some steroids present at very low levels. Liquid chromatography (LC) coupled to tandem MS (MS/MS) is increasingly being used as an analytical tool alternative to GC-MS procedures. There are several reports regarding AAS analysis using liquid chromatography–tandem mass spectrometry (LC-MS/MS) in human samples [15,16,17,18,19]. These methods are usually applied to urine samples for anti-doping analysis, since this matrix contains relatively high concentrations of drugs and/or their metabolites and its sampling is non-invasive. Nevertheless, classical urine sampling and handling requires storage and transportation at low and controlled temperatures, thus increasing the overall costs of analysis and the complexity of pre-analytical steps. Moreover, the possible occurrence of microorganisms in urine samples can alter AAS profiles, as it can trigger metabolism in stored samples [20,21]. Sample stability over time is indeed a crucial aspect, especially in anti-doping activities when delayed sample re-analysis is required, like in the case of the so-called “B-sample”, an aliquot of the sample originally supplied by the athlete, kept by the laboratory in charge and analysed as a confirmation if a prohibited substance is found in the original sample [22]. The purpose of this work is the development and comparison of two original urine microsampling and pretreatment techniques coupled to a fully validated LC-MS/MS method for the purpose of AAS analysis, with the aim of overcoming the current limitations arising from the use of large volumes of fluid samples and to develop new simplified procedures while maintaining high reliability and soundness of the resulting analytical data [23]. Thirteen representative AAS have been considered, namely nandrolone, 1-androstenedione, dehydroepiandrosterone (DHEA), testosterone, epitestosterone, dihydrotestosterone (DHT), methandrostenolone, norethandrolone, mesterolone, clostebol, stanozolol, fluoxymesterone and danazol (see Table 1 and Table 2 for structures).

In particular, dried urine spot (DUS) technique and the recently developed volumetric absorptive microsampling (VAMS) approach represent two innovative and promising alternatives to classical in-tube fluid sampling. Their full applicative potential is related to the numerous advantages they possess over traditional urine sampling: simplified and straightforward sample collection, enhanced compound stability, no requirement for controlled temperature transport and storage, overall cost savings. In addition to these common features, VAMS also provides an innovative, attractive way to sample accurate volumes of urine; the VAMS device was in fact initially engineered and developed for blood collection [24,25,26,27], but it can also be suitable for other biological matrices such as urine, plasma and oral fluid [26,27,28,29].

This is the first study to describe a comparative DUS/VAMS methodology coupled to LC-MS/MS detection, as a feasible and reliable strategy to assess free AAS concentrations in dried urine microsamples. DUS and VAMS process parameters have been investigated in order to develop effective and reliable protocols. Moreover, urine microsamples were compared with conventional fluid urine analysis, in order to assess correspondence of the obtained quantitative results. Finally, stability studies on dried microsamples were carried out and the results compared to those of classic sample cryopreservation.

The comparative analytical approach for dried urine microsamples presented herein aims at providing data useful to implement simple and rapid analytical protocols, with potential immediate applicability in sport drug testing scenarios and in neurotoxicity or neuroprotection studies.

## 2. Results and Discussion

### 2.1. Study of Sampling, Extraction and Separation Parameters

#### 2.1.1. Sampling Assays

The VAMS device is certified by the manufacturer for the reliable absorption of a fixed blood volume (10, 20 or 30 µL), and this performance level has been verified independently [30,31]. However, until now the device performance has not been extensively tested on other matrices, such as urine [32]. For this reason, a gravimetric approach was adopted to evaluate the accuracy and reproducibility of the absorbed urine volume. The VAMS device was weighed before and immediately after sampling; the difference (sampled weight) was divided by the mean density of the urine matrix as assessed by weighing 30 µL of urine sampled by pipetting (1.03 mg/µL) to obtain an estimate of the sampled volume. The resulting mean volume sampled by VAMS ± standard deviation (SD) was 30.06 ± 0.86 µL. Thus, volume accuracy was very good, with a slightly higher variability for VAMS (±0.86) than for pipetting (±0.25).

The effect of sample contact time was studied in the 1–30 s range. After 3 s, the sample volume absorbed onto the VAMS tip reached a plateau. For this reason, a safety margin was applied, and a 5-s sampling time was adopted.

Drying time was tested for both micromatrices. Under normal laboratory conditions, complete sample drying with constant weight was reached within 50 min for both DUS and VAMS. For DUS, it was possible to apply forced drying conditions by microwave (MW) irradiation. After extensive testing of both MW power (100–1000 W) and drying time (10–300 s), 800 W for 100 s was found to be the best compromise that does not cause significant analyte loss due to thermal instability. MW drying could not be applied to VAMS due to damage to the polymeric tip structure observed even at low potency. Forced VAMS drying was obtained by blowing filtered air, dried by a dehumidifier apparatus (30% RH, 0.25 kg/s flow rate), on the wet microsamples; this allowed to decrease drying time to less than 15 min. A precautionary drying time of 20 min was adopted. Details of drying condition optimisation results are reported in Supplementary Materials, Figures S1–S3.

#### 2.1.2. Extraction Procedure

The analytes were extracted from dried matrices using solvent extraction. Different pure solvents (acetonitrile, methanol, ethyl acetate, isopropanol) and solvent mixtures (also with water, buffers) were tested. The best combination of high extraction yields and good sample purification was obtained with pure methanol. A 500-µL volume was sufficient to obtain practically complete analyte extraction. For both matrices, simple vortexing, ultrasound-assisted extraction and microwave-assisted extraction were tested. Best results were obtained with ultrasound-assisted extraction, while vortexing produced noticeably lower extraction yields and microwave-assisted extraction tended to cause analyte loss (i.e., extraction yields decreased when increasing irradiation time). See Supplementary Materials Figures S4 and S5 for details of the extraction optimisation results and Table 3 for a comprehensive overview on optimised sampling, drying, storage and pretreatment protocols for urine VAMS and DUS samples.

### 2.2. LC-MS/MS Analysis and Method Validation

**Figure 1:**
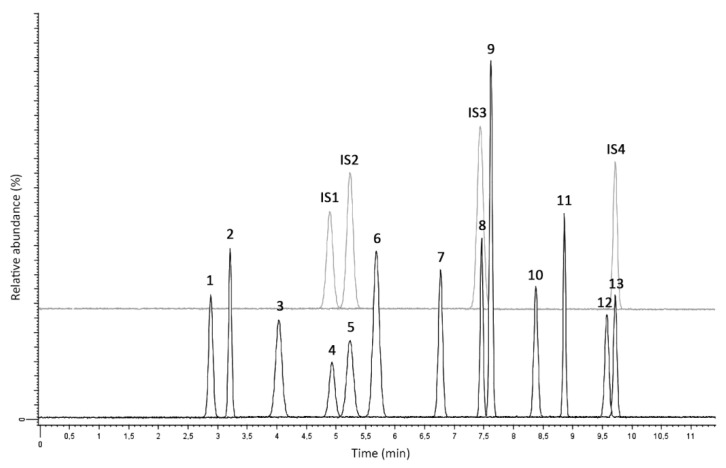
Liquid chromatography–tandem mass spectrometry (LC-MS/MS) chromatogram of a blank VAMS sample spiked with the analytes and ISs. 1: fluoxymesterone, 2: 1-androstenedione, 3: nandrolone, 4: dehydroepiandrosterone (DHEA), 5: testosterone, 6: epitestosterone, 7: clostebol, 8: dihydrotestosterone (DHT), 9: methandrostenolone, 10: norethandrolone, 11: mesterolone, 12: danazol, 13: stanozolol.

The LC system was chosen for maximum flexibility. For this reason, a high-resolution (3.5 µm), narrow-bore (2.1 mm) stationary phase was used, without reaching UHPLC-grade performance: in this way, common HPLC apparatuses can be used, while achieving complete resolution of all analytes in a relatively short time (10 min). The LC-MS/MS chromatogram of a blank VAMS sample spiked with the analytes and internal standards (ISs) is shown in Figure 1.

#### 2.2.1. Linearity and Selectivity

Linearity parameters are reported in Table 4. As one can see, wide linearity ranges were achieved on both matrices, with very satisfactory correlation coefficients (r^2^ ≥ 0.9990).

Selectivity was assessed by injecting samples prepared from steroid-depleted urine from volunteers. None of these samples produced a peak corresponding to the analytes with areas higher than the limit of detection (LOD). Selectivity was thus considered satisfactory.

#### 2.2.2. Absolute Recovery and Precision

Complete results from the extraction yield and precision assays are shown in Table 5. As one can see, absolute recovery was higher than 77.1% and 70.3% for VAMS and DUS, respectively, with relative standard deviation (RSD) values in the 5.3–7.0% range for VAMS and in the 5.8–7.5% range for DUS, as regards the considered analytes. Generally speaking, both absolute recovery and precision were better for VAMS than for DUS: the higher precision of VAMS was marginal, without statistical significance, on the contrary absolute recovery results were consistently higher for VAMS (up to +11%, mean value), with common trends for all the 13 analytes.

#### 2.2.3. Matrix Effect and Stability

**Figure 2:**
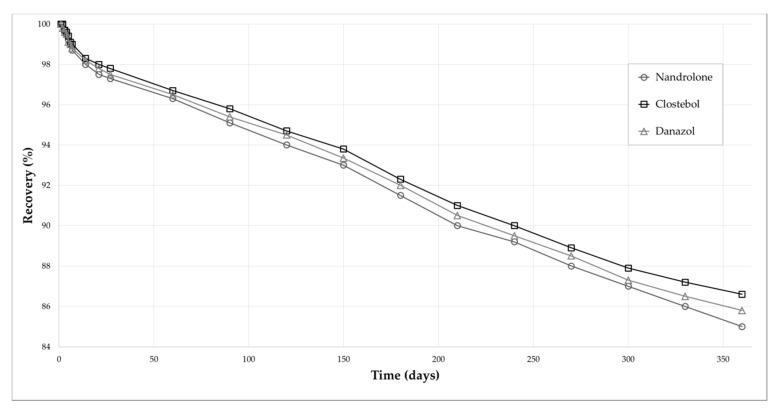
Stability results from blank spiked urine VAMS stored at RT.

Matrix effect, expressed as percentage relative error (RE%), was low for both matrices (<11.9%, Table 6) at all concentration levels. Long-term stability assays were carried out on both dried matrices, also keeping in mind that “B samples” may have to be re-analysed even months after the original sampling time. The samples were stored at RT, in the dark and in sealed polyethylene envelopes with a desiccant. IS-normalised analyte recovery from VAMS was always higher than 88% after 3 months, and higher than 82% after 1 year; and 85% and 70% for DUS, respectively (Table 6). The outstanding results of analyte stability over 1-year storage in VAMS can also be visualised graphically in Figure 2 for three analytes (nandrolone, clostebol, danazol).

The method was applied to real samples from subjects who admitted to taking one or more AAS for doping purposes. After urine sampling, both DUS and VAMS were prepared from the same sample.

**Figure 3:**
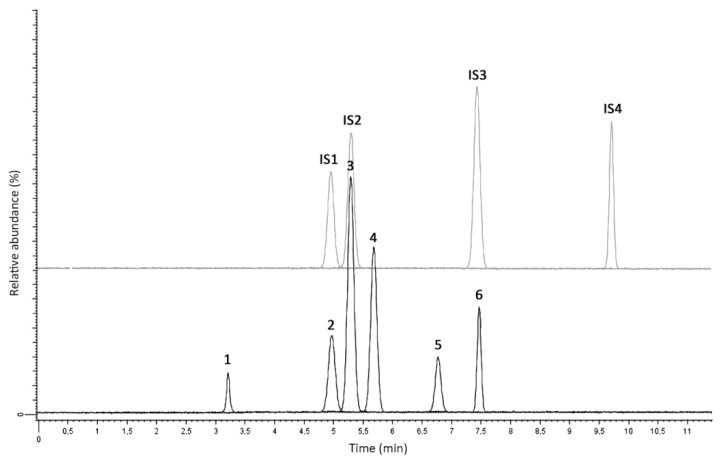
LC-MS/MS chromatogram from a real urine sample spiked with ISs. 1: 1-androstenedione, 2: DHEA, 3: testosterone, 4: epitestosterone, 5: clostebol, 6: DHT.

The results obtained from these analyses were quite satisfactory; the analyte(s) were found in all samples, without appreciable interference. Complete analysis results can be found in Table 7. An example of chromatogram from the analysis of DUS obtained from a real urine sample is shown in Figure 3.

Some real urine samples were spiked with the corresponding analyte(s) and ISs, then DUS and VAMS were prepared and analysed. The percentage recovery of the added analyte(s) was calculated to assess accuracy. Good accuracy was found, in the 89–97% range for all AAS; mean recovery was 93% (RSD < 5.4%).

### 2.3. Comparison with Fluid Samples

**Figure 4:**
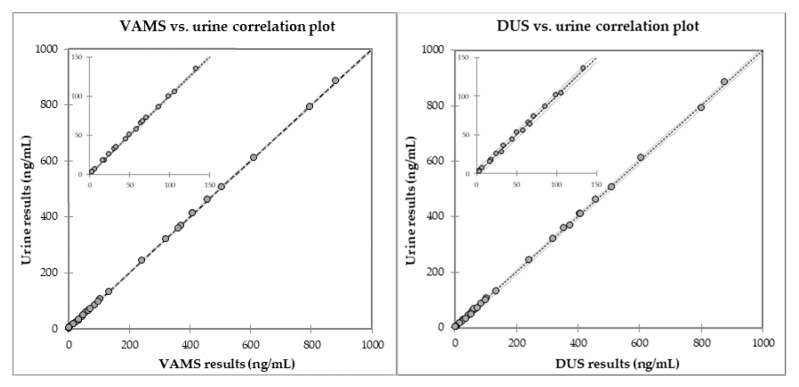
Statistical correlation plots for VAMS vs. urine and DUS vs. urine result comparison.

Fluid urine samples corresponding to the analysed DUS and VAMS microsamples were also analysed to assess the reliability of the new microsampling methods. The comparison was carried out by building the DUS vs. urine and VAMS vs. urine curves by means of the least squares method and evaluating the respective linearity coefficients and Passing–Bablok regression. Linear regression plots can be found in Figure 4 and the corresponding data are reported in Supplementary Materials Table S2. As can be seen, very good linearity was found, also with limited variance. Values of slope and intercept were also evaluated, as indicators of agreement between, and lack of bias of, the compared measures. As shown in Table S2, Passing–Bablok regression produced comparison slope coefficients very close to the unity (from 0.9942 to 1.0131), with negligible intercepts and regression coefficients higher than 0.9991. Both microsampling methods can thus be considered valid alternatives to the fluid urine sample.

## 3. Materials and Methods 

### 3.1. Chemicals and Solutions

Certified analytical standards of testosterone, mesterolone, danazol, DHT, methandrostenolone, and clostebol as pure powders; standard solutions at the concentration of 1 mg/mL in methanol of epitestosterone, 1-androstenedione, DHEA, norethandrolone, nandrolone, fluoxymesterone, stanozolol and ISs as stable isotope-labelled analogues solutions at 100 µg/mL in methanol of DHEA-D5 (IS1), testosterone-D3 (IS2), DHT-D3 (IS3) and stanozolol-D3 (IS4) were acquired from Cerilliant (Round Rock, TX, USA). Methanol and acetonitrile for LC-MS (≥99.9%) and formic acid for LC-MS (98–100%) were obtained from Sigma-Aldrich (Saint Louis, MO, USA). Ultrapure-grade water (18.2 MΩ cm) was obtained in-house from a Millipore (Milford, MA, USA) MilliQ Gradient apparatus. Stock solutions of individual powder compounds (1 mg/mL) were prepared in methanol. Working standard solutions in methanol of all analytes and IS were prepared daily from stock solutions and stored at −20 °C in amber glass vials.

### 3.2. LC-MS/MS System

The LC-MS/MS system consisted of a Waters (Milford, MA, USA) Alliance e2695 chromatographic pump coupled to a Waters Micromass Quattro Micro triple quadrupole mass spectrometer, through an electrospray ionization source operating in positive mode (ESI+). Data acquisition was performed in multiple reaction monitoring mode (MRM) with the following optimised parameters: ionisation source voltage, 4.00 kV; ionization source temperature, 120 °C; desolvation temperature, 150 °C; desolvation gas flow (nitrogen), 750 L/h; argon was used as collision gas. The dwell time per channel was set to 300 ms for each analyte and IS. Precursor and product ions (both quantifier and qualifier ions), cone voltage and collision energy values optimised ad hoc for each analyte and IS are reported in Table 8. Data were processed using Waters MassLynx 4.1 software.

The stationary phase used for chromatographic separations is a Restek (Bellefonte, PA, USA) Raptor C18 column (50 × 2.1 mm I.D., 2.7 µm particles), equipped with a C18 guard column (5 × 2.1 mm I.D., 2.7 µm particles). The mobile phase consisted of a mixture of 0.1% formic acid in acetonitrile (component A) and 0.1% formic acid in water (component B) under the following automatic composition gradient program: 0 min, 30% A; 1.50 min, 30% A; 3.00 min, 70% A; 10.00 min, 70% A; 12.00 min, 30% A; 15 min, 30% A. Complete analyte separation took less than 10 min, while the full chromatographic run lasted 15 min, including column reconditioning. Autosampler-injected volume was 10 µL and the flow rate was kept constant at 300 µL/min.

Each of the four ISs was used for the quantification of those analytes with similar retention times: DHEA-D5 (IS1) was used for the quantification of fluoxymesterone, 1-androstenedione, nandrolone and DHEA; testosterone-D3 (IS2) was used for testosterone, epitestosterone and clostebol; DHT-D3 (IS3) was used for DHT, methandrostenolone and norethandrolone; stanozolol-D3 (IS4) was used for mesterolone, danazol and stanozolol.

### 3.3. Microsample Collection and Pretreatment

The proposed miniaturised sampling strategies were applied to blank urine coming from healthy volunteers for method development and validation. Since some analytes (testosterone, epitestosterone, DHT and DHEA) are endogenous, steroid-depleted urine was prepared according to the method by AbuRuz et al. [33], with some modifications. A urine pool (100 mL) from female volunteers was stirred with charcoal (0.5 g) and dextran (0.05 g) for 8 h at 4 °C. The sample was then centrifuged at 4000× *g* for 10 min, vacuum filtered through paper and stored at −20 °C until use. Blank samples were analysed to ascertain that residual steroid amounts were lower than the respective LOD values. Real urine samples were obtained from self-reported AAS users.

Aliquots of 90 µL of urine were fortified with 10 µL of a standard solution containing the analytes at known concentrations (and ISs at constant concentrations). The obtained spiked urine samples were then subjected to microsampling, pretreatment and LC-MS/MS analysis.

#### 3.3.1. Volumetric Absorptive Microsampling

30-µL VAMS devices, commercialised under the Mitra^®^ trade name, were obtained from Neoteryx (Torrance, CA, USA). Sampling is performed by placing the polymeric tip end in contact with the urine sample surface with an angle of 45° for a total time of 5 ± 1 s, in order to allow uniform adsorption. The devices are then placed in the designated clam-shell packaging which also acts as a support during the forced drying phase, which was carried out by blowing filtered air, dried by a dehumidifier apparatus (30% RH, 0.25 kg/s flow rate), on the wet microsamples for 20 min. The enclosure was then stored in polyethylene resealable bags containing desiccant silica gel packets. At the time of analysis, the dried tip is detached from the sampler body and placed inside a vial. Analyte desorption from the polymeric matrix is performed with 500 µL of methanol under ultrasonication at RT for 5 min. The extract is quantitatively transferred into a new vial and evaporated to dryness under a gentle N_2_ stream using a Sigma-Aldrich Mini-Vap evaporator. The residue is finally re-dissolved with 100 µL of methanol and analysed by LC-MS/MS without the need for further treatment.

#### 3.3.2. Dried Urine Spot Microsampling

Whatman (Maidstone, UK) FTA DMPK-C cards were used for DUS collection, where 10 µL of urine was pipetted into the centre of pre-marked circles. DUS were subjected to forced drying by MW (800 W for 100 s) and then stored in polyethylene resealable bags containing desiccant silica gel packets until pretreatment and analysis. At the time of analysis, a 10-mm diameter circle was punched out from the card with a puncher and placed into a vial with 500 µL of methanol, extracted under ultrasonication at RT for 5 min and centrifuged at 4000 rpm for 5 min at 4 °C. The supernatant was brought to dryness under a gentle N_2_ stream, re-dissolved with 100 µL of methanol and analysed by LC-MS/MS without the need for further treatment. 

### 3.4. Method Validation

#### 3.4.1. Calibration Curves

Aliquots of 10 µL of analyte standard solutions at seven different concentrations, containing the ISs at constant concentrations, were added to blank matrices before sampling. The resulting spiked DUS or VAMS samples were subjected to the previously described pretreatment procedures and finally injected into the LC-MS/MS system. The procedure was carried out in triplicate for each concentration. The analyte/IS peak area ratios obtained were plotted against the corresponding concentrations of the analytes (expressed as ng/mL) and the calibration curves set up by means of the least-square method. The values of limit of quantitation (LOQ) and LOD were calculated according to the International Conference on Harmonisation (ICH) guidelines [25] as the analyte concentrations, which give rise to peaks whose heights are 10 and 3 times the baseline noise, respectively. Method detection limits (MDL) values have been calculated on 7 replicates according to Environmental Protection Agency’s (EPA) guidelines, as *MDL = t^(n − 1, 1 – α = 0.99)^ S*, where *t^(n − 1, 1 – α = 0.99)^* is the Student’s t value appropriate for a single-tailed 99th percentile t statistic and a standard deviation estimate with *n* − 1 degrees of freedom, and *S* is the standard deviation of the replicate spiked sample analyses.

#### 3.4.2. Absolute Recovery and Precision Assays

Absolute recovery and precision were evaluated by adding known amounts of the analytes (at three different concentrations representative of each calibration curve) to blank DUS or VAMS, then subjecting the mixtures to the sample pretreatment and injecting them into the LC-MS/MS system. 

The analyte peak areas were compared to those obtained by injecting standard solutions at the same theoretical concentrations and absolute recovery was calculated. The assays described above were repeated six times within the same day to obtain intraday precision (repeatability) and six times over six different days to obtain interday precision (intermediate precision), both expressed as relative standard deviation (RSD%).

#### 3.4.3. Matrix Effect

Matrix effect was evaluated by adding known amounts of the analytes (at 3 different concentrations, corresponding to the lower limit, a middle point and a high value of each calibration curve) to pretreated blank matrices, immediately before injection. The peak areas obtained from these assays were compared to those of the standard solutions and the corresponding percentage relative error (RE%) values were calculated.

#### 3.4.4. Selectivity

Blank samples from six different volunteers were subjected to the pretreatment procedure and injected into the HPLC-MS/MS system. The resulting chromatograms were checked for possible interference from endogenous compounds. The acceptance criterion was no interfering peak higher than an analyte peak corresponding to its LOD. 

#### 3.4.5. Stability

Stability was tested in methanolic stock solutions for each analyte and IS stored for 30 days at −80 °C, by comparison with those freshly purchased (*n* = 3). 

To evaluate analyte stability in the dried matrices, blank samples were spiked with the analytes and the ISs and then analysed at regular intervals until 12 months from sampling. During this time, DUS and VAMS were stored at RT, in a dark and dry place. The results obtained after IS normalisation were compared to those of spiked samples analysed immediately after spotting and drying.

#### 3.4.6. Analysis and Comparison with Urine Samples from Real Cases

Fluid urine aliquots of 100 µL, taken from volunteers or users, were spiked with 5 μL of standard solution containing analytes and/or ISs at known concentrations; the vial was mixed by vortex agitation. The solution was then subjected to microextraction by packed sorbent (MEPS) pretreatment in an SGE Analytical Science (Melbourne, VIC, Australia) C8 barrel insert and needle (BIN) assembly set up in an SGE eVol XR digital analytical syringe apparatus. The BIN was activated with 100 µL of methanol drawn and discarded 3 times, then conditioned 3 times with 100 µL of water. The sample was loaded in the BIN with 15 draw/discharge cycles at a speed of 5 µL/s; BIN washing was then performed with 100 µL of 25 mM, pH 7.4 phosphate buffer at 20 µL/s and finally the analyte and the IS were eluted with 250 µL of methanol at 5 µL/s (single cycle). The eluate was dried, re-dissolved in 50 µL of solvent A/solvent B (10/90, *V*/*V*) mixture and analysed by LC-MS/MS. The results obtained from fluid urine samples were then compared to those obtained from the corresponding DUS and VAMS samples.

#### 3.4.7. Accuracy

Accuracy was evaluated by means of recovery assays as described by the International Conference on Harmonisation (ICH) [34]. Known amounts of the analytes at three levels (the lower limit, the middle point and a high value of each calibration curve) and of the ISs at constant levels were added to real samples whose analyte content was already known, since they had been already analysed. The spiked samples were then pretreated and analysed to obtain the mean recovery (%) of the added analytes. These assays were repeated three times during the same day to calculate the corresponding SD data.

## 4. Conclusions

Two innovative microsampling methods, based on DUS and VAMS, were developed for the purpose of anti-doping analysis. These dried micromatrices have been demonstrated to be much more practical than traditional urines: they can be stored for up to 12 months at RT without losing more than 18% (for VAMS) and more than 30% (for DUS) of the original analyte content. Moreover, being miniaturised, they occupy much less shelf space and storage equipment and require just minute amounts of solvents for extraction. Finally, the validation and comparison assays have provided ample assurance that these micromatrix sampling approaches provide performances comparable to, and often even better than, those of the corresponding fluid urine matrix, in terms of absolute recovery (recovery > 77.1% for VAMS, >70.3% for DUS), precision (RSD < 7.1% for VAMS, <7.6% for DUS), matrix effect (RE < 9.3% for VAMS, <11.9% for DUS) and accuracy (89–97% range); analyte levels found in real samples were not statistically different.

Comparison with existing methods (Supplementary Materials Table S1) shows that most of them require time-consuming pretreatment steps, often including sample derivatisation. Many methods are also not fully validated, and those that are validated have analytical performances similar to, or worse than, the proposed method in terms of absolute recovery and matrix effect. Some methods achieve outstanding sensitivity, but of course using higher sample volumes; however, they still have the inconvenience of dealing with fluid urine samples in terms of handling, shipping, storage and related expenses.

Neither of the two dried matrix approaches can be said to be significantly better than the other and each one can be personalised and used according to the needs of the moment, available equipment and personnel, and local law. Nevertheless, the thorough comparative study carried out within this research highlighted overall better performances of VAMS over DUS, especially in terms of long-term stability, but both are reliable, cheap and convenient.

## Figures and Tables

**Table 1:**
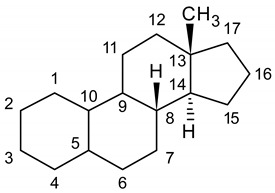
Chemical structures of the analytes (first part).

Carbon N.	Nandrolone	1-Androstenedione	DHEA	Testosterone	Epitestosterone	DHT	Methandrostenolone
1	-H_2_	-H	-H_2_	-H_2_	-H_2_	-H_2_	-H
2	-H_2_	-H	-H_2_	-H_2_	-H_2_	-H_2_	-H
1,2		-C=C-					-C=C-
3	=O	=O	-OH β	=O	=O	=O	=O
2,3							
4	-H	-H_2_	-H_2_	-H	-H	-H_2_	-H
5		-H α				-H α	
4,5	-C=C-			-C=C-	-C=C-		-C=C-
6	-H_2_	-H_2_	-H	-H_2_	-H_2_	-H_2_	-H_2_
5,6			-C=C-				
9	-H α	-H α	-H α	-H α	-H α	-H α	-H α
10	-H β	-CH_3_ β	-CH_3_ β	-CH_3_ β	-CH_3_ β	-CH_3_ β	-CH_3_ β
11	-H_2_	-H_2_	-H_2_	-H_2_	-H_2_	-H_2_	-H_2_
17	-OH β	=O	=O	-OH β	-OH α	-OH β	-OH β, -CH_3_ α

**Table 2:**
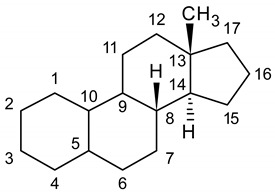
Chemical structures of the analytes (second part).

Carbon N.	Norethandrolone	Mesterolone	Clostebol	Stanozolol	Fluoxymesterone	Danazol
1	-H_2_	-CH_3_ α	-H_2_	-H_2_	-H_2_	-H_2_
2	-H_2_	-H_2_	-H_2_		-H_2_	
3	=O	=O	=O		=O	
2,3				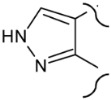		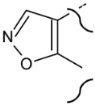
4	-H	-H_2_	-Cl	-H_2_	-H	-H
5		-H α		-H α		
4,5	-C=C-		-C=C-		-C=C-	-C=C-
6	-H_2_	-H_2_	-H_2_	-H_2_	-H_2_	-H_2_
9	-H α	-H α	-H α	-H α	-F α	-H α
10	-H β	-CH_3_ β	-CH_3_ β	-CH_3_ β	-CH_3_ β	-CH_3_ β
11	-H_2_	-H_2_	-H_2_	-H_2_	-OH β	-H_2_
17	-OH β, -CH_2_CH_3_ α	-OH β	-OH β	-OH β, -CH_3_ α	-OH β, -CH_3_ α	-OH β, -C≡CH α

**Table 3:** Optimised sampling and pretreatment for VAMS and DUS.

Matrix	Sampling	Drying	Storage	Extraction
VAMS	Direct contact	Forced drying (air blowing, room temperature (RT), 20 min)	Dedicated clamshell + resealable bag with desiccant (RT)	500 µL methanol, 5 min ultrasonication, drying (N_2_ stream), re-dissolution with 100 µL methanol
DUS	Pipetting	Forced drying (MW, 80W, 100 s)	Resealable bag with desiccant (RT)	500 µL methanol, 5 min ultrasonication, centrifugation (4000 rpm, 5 min, 4 °C), drying (N_2_ stream), re-dissolution with 100 µL methanol

**Table 4:** Linearity, limit of quantification (LOQ), limit of detection (LOD), method detection limit (MDL).

Analyte	Linearity Range, ng/mL	*r* ^2^	LOQ, ng/mL	LOD, ng/mL	MDL, ng/mL
Nandrolone	1.0–750.0	0.9990	1.0	0.3	0.2
1-Androstenedione	1.5–500.0	0.9993	1.5	0.5	0.5
DHEA	1.5–750.0	0.9992	1.5	0.5	0.5
Testosterone	1.0–750.0	0.9995	1.0	0.3	0.2
Epitestosterone	1.0–750.0	0.9990	1.0	0.3	0.2
DHT	1.5–750.0	0.9992	1.5	0.5	0.5
Methandrostenolone	1.5–500.0	0.9994	1.5	0.5	0.5
Norethandrolone	1.5–500.0	0.9996	1.5	0.5	0.5
Mesterolone	1.5–500.0	0.9990	1.5	0.5	0.5
Clostebol	1.5–500.0	0.9994	1.5	0.5	0.5
Stanozolol	1.5–750.0	0.9997	1.5	0.5	0.5
Fluoxymesterone	1.5–500.0	0.9997	1.5	0.5	0.5
Danazol	1.5–750.0	0.9995	1.5	0.5	0.5

Abbreviations: LOD, limit of detection; LOQ, limit of quantitation; MDL, method detection limit.

**Table 5:** Extraction yield and precision.

Analyte	Concentration (ng/mL)	Extraction Yield ±SD, % ^a^		Precision, RSD% ^a^			
				Repeatability		Intermediate Precision	
		DUS	VAMS	DUS	VAMS	DUS	VAMS
Nandrolone	1.0	73.4 ± 2.0	81.0 ± 3.7	7.1	6.5	6.6	6.2
	375.0	72.1 ± 1.4	80.5 ± 2.9	6.9	6.0	6.4	5.7
	750.0	72.5 ± 1.5	82.3 ± 1.6	6.1	5.8	5.7	5.4
1-Androstenedione	1.5	71.9 ± 3.3	82.6 ± 3.4	7.4	7.0	7.0	6.7
	250.0	73.4 ± 2.5	85.3 ± 2.0	7.0	6.5	6.4	6.1
	500.0	75.6 ± 2.7	86.2 ± 1.9	6.5	6.2	6.1	5.7
DHEA	1.5	84.3 ± 4.3	90.1 ± 3.7	7.4	7.0	7.0	6.9
	375.0	86.7 ± 2.6	90.2 ± 3.4	7.2	7.0	6.6	6.4
	750.0	85.4 ± 2.8	92.0 ± 2.7	7.0	6.8	6.3	6.1
Testosterone	1.0	71.6 ± 3.4	77.3 ± 3.3	7.3	7.0	6.9	6.6
	375.0	73.1 ± 2.4	78.4 ± 3.8	6.5	6.4	6.2	5.9
	750.0	75.9 ± 2.6	80.3 ± 2.4	6.2	5.9	6.0	5.3
Epitestosterone	1.0	71.3 ± 4.1	79.1 ± 4.6	7.1	6.8	6.8	6.3
	375.0	75.5 ± 2.8	79.1 ± 3.1	6.3	6.2	6.4	5.8
	750.0	76.4 ± 3.5	82.0 ± 2.8	6.2	5.8	5.8	5.5
DHT	1.5	75.0 ± 2.7	81.6 ± 2.6	7.3	7.0	7.0	6.9
	375.0	78.1 ± 2.4	80.4 ± 2.7	7.2	6.9	6.8	6.6
	750.0	78.5 ± 1.9	82.7 ± 1.9	6.3	6.7	6.0	6.3
Methandrostenolone	1.5	74.8 ± 3.2	83.2 ± 4.0	7.0	6.7	6.5	6.2
	250.0	78.6 ± 2.4	85.9 ± 3.5	6.6	6.3	6.3	6.0
	500.0	76.3 ± 1.8	84.6 ± 2.8	6.3	6.1	5.9	5.7
Norethandrolone	1.5	74.9 ± 3.6	77.3 ± 2.6	7.0	6.6	6.6	6.2
	250.0	73.5 ± 2.9	77.5 ± 2.9	6.6	6.3	6.2	5.9
	500.0	74.0 ± 2.7	79.3 ± 1.9	6.2	5.9	5.8	5.5
Mesterolone	1.5	73.2 ± 2.6	80.8 ± 3.3	7.5	7.0	7.1	6.8
	250.0	77.2 ± 2.5	81.3 ± 3.6	7.4	6.7	7.0	6.4
	500.0	78.3 ± 1.7	79.9 ± 2.8	6.9	6.5	6.4	6.2
Clostebol	1.5	73.6 ± 4.0	81.5 ± 3.1	7.4	6.9	7.0	7.0
	250.0	75.7 ± 3.6	82.5 ± 2.7	7.0	6.8	6.8	6.6
	500.0	76.6 ± 2.9	83.8 ± 3.0	6.9	6.6	6.3	6.3
Stanozolol	1.5	70.4 ± 3.2	82.7 ± 3.3	5.7	5.4	6.3	6.0
	375.0	71.8 ± 3.5	85.2 ± 2.5	5.6	5.2	6.1	5.8
	750.0	74.4 ± 2.9	86.7 ± 2.7	5.1	5.0	5.7	5.5
Fluoxymesterone	1.5	71.1 ± 3.2	80.6 ± 3.4	7.2	7.0	6.9	6.7
	250.0	74.4 ± 2.7	83.4 ± 2.8	7.1	6.9	6.9	6.6
	500.0	75.1 ± 2.6	83.7 ± 1.9	7.0	6.8	6.5	6.3
Danazol	1.5	72.8 ± 3.4	80.8 ± 4.6	6.6	6.5	6.3	6.2
	375.0	70.6 ± 2.6	81.3 ± 4.3	6.9	6.5	6.4	6.1
	750.0	71.6 ± 2.8	84.5 ± 2.6	6.4	6.2	6.0	5.8
IS1	20.0	82.7 ± 2.5	89.7 ± 1.8	4.2	4.6	3.8	3.4
IS2	20.0	80.9 ± 1.9	88.4 ± 1.8	4.5	4.8	3.6	3.2
IS3	20.0	83.5 ± 2.0	88.0 ± 2.0	4.3	4.7	3.8	3.5
IS4	20.0	85.7 ± 2.2	90.1 ± 1.9	4.3	4.8	3.8	3.1

^a^*n* = 6.

**Table 6:** Matrix effect and stability.

Analyte	Concentration (ng/mL)	Matrix Effect, %RE ^a^		Stability, % Recovery ^a,b^			
				3 Months		1 Year	
		DUS	VAMS	DUS	VAMS	DUS	VAMS
Nandrolone	1.0	11.3	9.2	91	92	73	84
	375.0	11.2	8.3	90	90	75	85
	750.0	9.0	8.3	92	93	77	87
1-Androstenedione	1.5	10.3	8.2	88	90	72	88
	250.0	9.3	7.5	89	90	73	87
	500.0	10.1	6.6	88	89	75	88
DHEA	1.5	9.8	8.8	89	89	75	83
	375.0	9.5	6.9	90	91	77	84
	750.0	8.2	6.8	90	90	77	86
Testosterone	1.0	6.2	8.7	87	90	73	84
	375.0	7.2	6.1	88	91	74	85
	750.0	6.2	7.1	89	91	76	85
Epitestosterone	1.0	8.0	7.0	87	90	73	87
	375.0	7.0	5.0	89	92	73	88
	750.0	63	4.2	90	93	75	88
DHT	1.5	7.9	5.1	89	91	74	84
	375.0	5.9	3.2	90	91	74	86
	750.0	5.4	3.2	90	94	76	87
Methandrostenolone	1.5	9.8	9.2	88	90	73	85
	250.0	10.1	9.2	89	90	75	86
	500.0	9.1	8.2	90	90	74	86
Norethandrolone	1.5	9.1	8.8	87	91	73	87
	250.0	9.2	7.9	88	93	74	88
	500.0	8.3	6.6	90	92	75	88
Mesterolone	1.5	10.4	7.3	87	90	74	86
	250.0	8.5	7.2	88	89	73	87
	500.0	6.5	7.3	88	90	74	85
Clostebol	1.5	11.8	8.1	88	90	74	84
	250.0	11.4	8.5	90	90	75	87
	500.0	9.0	6.7	90	93	75	87
Stanozolol	1.5	10.3	6.8	86	90	73	84
	375.0	8.2	4.7	87	91	74	85
	750.0	7.3	4.6	89	91	75	84
Fluoxymesterone	1.5	9.4	7.6	87	90	72	84
	250.0	9.4	7.6	89	92	72	84
	500.0	7.3	6.3	91	93	73	86
Danazol	1.5	10.4	8.9	88	91	73	84
	375.0	9.3	6.3	89	91	71	86
	750.0	8.2	5.0	90	92	72	86

^a^*n* = 6, mean value. ^b^ Percentage recovery vs. the same spiked samples analysed immediately.

**Table 7:** Results of method application to real samples.

Subject n.	Matrix	Concentration Found, ng/mL ^a^				Other Identified AAS (Concentration, ng/mL)
		Testosterone	Epitestosterone	DHT	DHEA	
	Urine	106.1	31.0	64.6	321.5	Clostebol (6.8)
1	DUS	104.3	27.6	65.9	317.9	Clostebol (7.4)
	VAMS	105.4	33.1	65.3	320.4	Clostebol (6.5)
	Urine	58.2	24.6	18.2	412.6	Nandrolone (3.6)
2	DUS	55.9	25.9	17.5	409.6	Nandrolone (3.0)
	VAMS	57.6	25.5	17.9	410.5	Nandrolone (3.5)
	Urine	368.4	66.9	85.9	359.6	/
3	DUS	374.9	63.2	86.9	353.2	/
	VAMS	370.6	67.3	86.5	361.6	/
	Urine	612.3	44.8	49.9	245.1	Stanozolol (98.6)
4	DUS	605.1	44.0	53.6	242.3	Stanozolol (101.2)
	VAMS	610.3	45.2	50.5	243.1	Stanozolol (99.3)
	Urine	71.1	33.4	16.8	133.4	Clostebol (3.2)
5	DUS	74.3	36.1	15.3	135.6	Clostebol (3.8)
	VAMS	72.0	35.0	17.3	135.0	Clostebol (3.0)

^a^*n* = 3.

**Table 8:** MRM transitions, cone voltage, collision energy and retention time for each analyte and IS.

Analyte	Q1 (*m*/*z*)	Q3 (*m*/*z*) Quantifier	Q3 (*m*/*z*) Qualifier	Cone Voltage (V)	Collision Energy (eV)	*t*_R_ (min)
Nandrolone	275.0	109.0	239.1	25	25	4.07
1-Androstenedione	287.5	97.1	109.1	29	21	3.22
DHEA	289.1	271.2	213.2	29	21	4.91
Testosterone	289.5	109.2	97.1	25	25	5.25
Epitestosterone	289.5	109.2	97.1	25	25	5.19
DHT	291.4	121.0	255.2	25	25	7.48
Methandrostenolone	301.5	121.2	283.2	25	25	7.63
Norethandrolone	303.2	285.0	267.0	25	25	8.38
Mesterolone	305.0	269.0	229.2	34	16	8.87
Clostebol	323.0	143.0	131.0	25	25	6.76
Stanozolol	329.5	81.1	107.1	55	31	9.72
Fluoxymesterone	337.0	241.0	131.1	25	25	2.88
Danazol	338.2	148.0	109.1	25	25	9.49
IS1	292.0	112.0	-	25	25	4.91
IS2	277.2	219.2	-	25	25	5.25
IS3	294.0	258.0	-	25	25	7.48
IS4	294.1	276.2	-	29	21	9.72

## Supplementary Materials

The following are available online, Figure S1: Natural drying time assay of DUS and VAMS, Figure S2: Surface response graph for the microwave drying time assay of DUS, Figure S3: Forced drying time assay of VAMS, Figure S4: Extraction yield and purification assay results: extraction solvent comparison, Figure S5: Extraction yield and purification assay results: extraction means comparison. MAE, microwave-assisted extraction; UAE, ultrasound-assisted extraction, Table S1: Performance comparison of existing methods with the proposed method for anabolic steroid analysis, Table S2: Passing-Bablok regression parameters.
