# Supplementary material for: Dried Urine Microsampling Coupled to Liquid Chromatography—Tandem Mass Spectrometry (LC–MS/MS) for the Analysis of Unconjugated Anabolic Androgenic Steroids
Source: Molecules. 2020 Jul 14;25(14):3210. doi: 10.3390/molecules25143210 (PMC7397045; doi:10.3390/molecules25143210)
Supplement: Supplementary file 1 [file molecules-25-03210-s001.pdf]

molecules

Supplementary Material

Dried Urine Microsampling Coupled to Liquid Chromatography—Tandem Mass Spectrometry (LC–MS/MS) for the Analysis of Unconjugated Anabolic Androgenic Steroids

Michele Protti[1], Camilla Marasca[1], Marco Cirrincione[1], Angelo E. Sberna[2], Roberto Mandrioli[3], Laura Mercolini[1,*]

[1] Research group of Pharmaco-Toxicological Analysis (PTA Lab), Department of Pharmacy and Biotechnology (FaBiT), Alma Mater Studiorum - University of Bologna, Bologna, Italy
[2] Sport Medicine, Enna Local Health Unit, Enna, Italy
[3] Department for Life Quality Studies, Alma Mater Studiorum - University of Bologna, Rimini, Italy
* Correspondence: laura.mercolini@unibo.it; Tel.: +39 051 209 9726

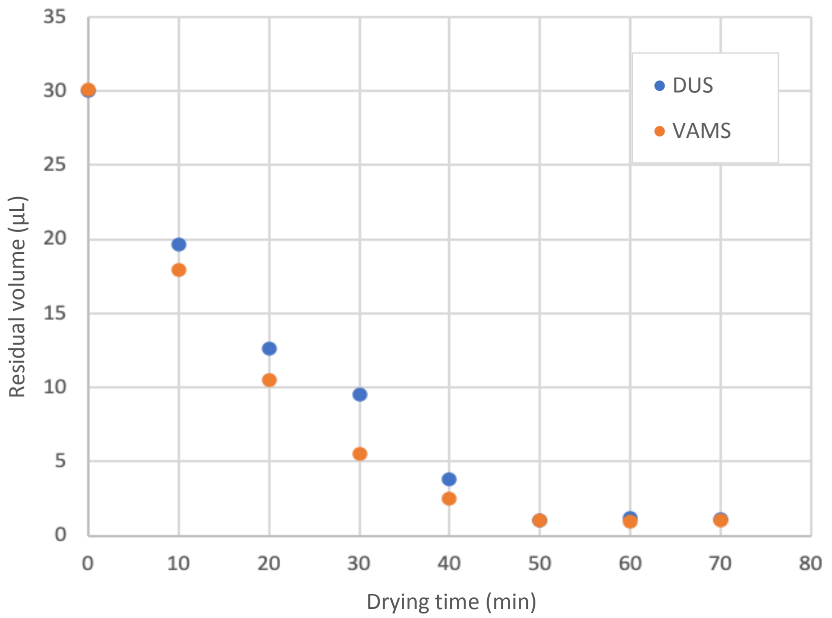

Figure S1. Natural drying time assay of DUS and VAMS.

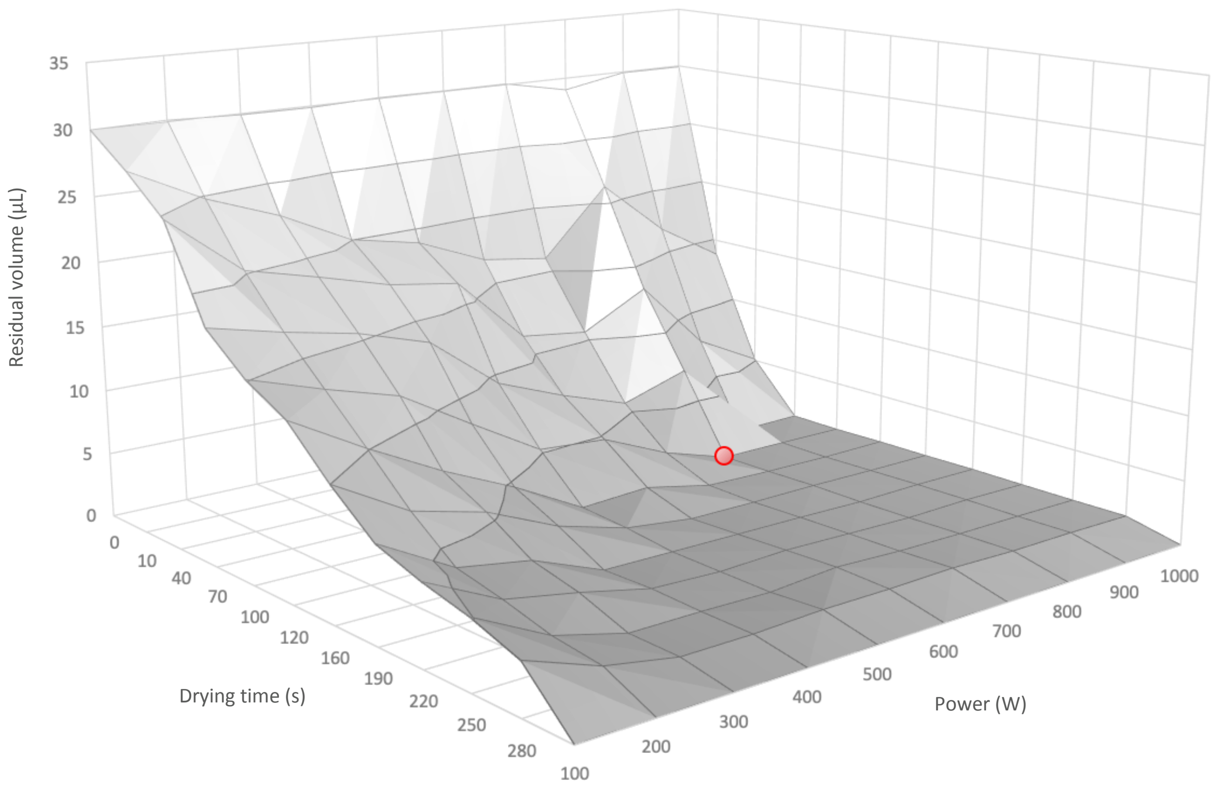

Figure S2. Surface response graph for the microwave drying time assay of DUS.

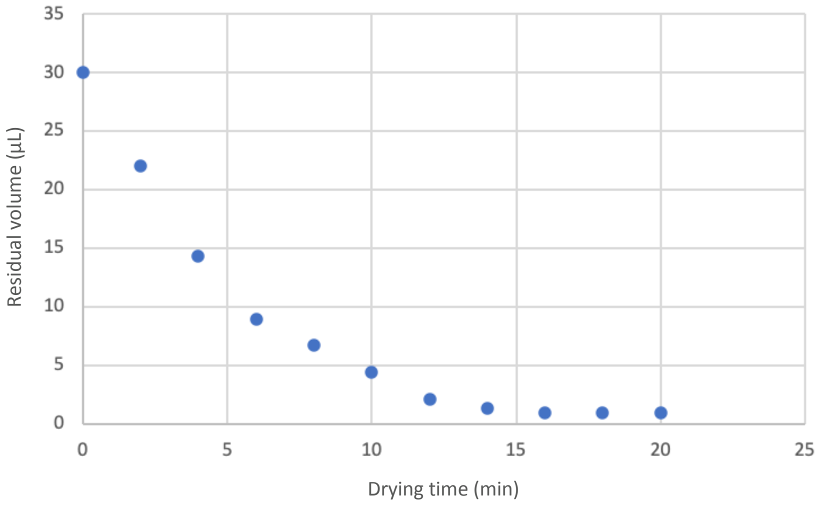

Figure S3. Forced drying time assay of VAMS.

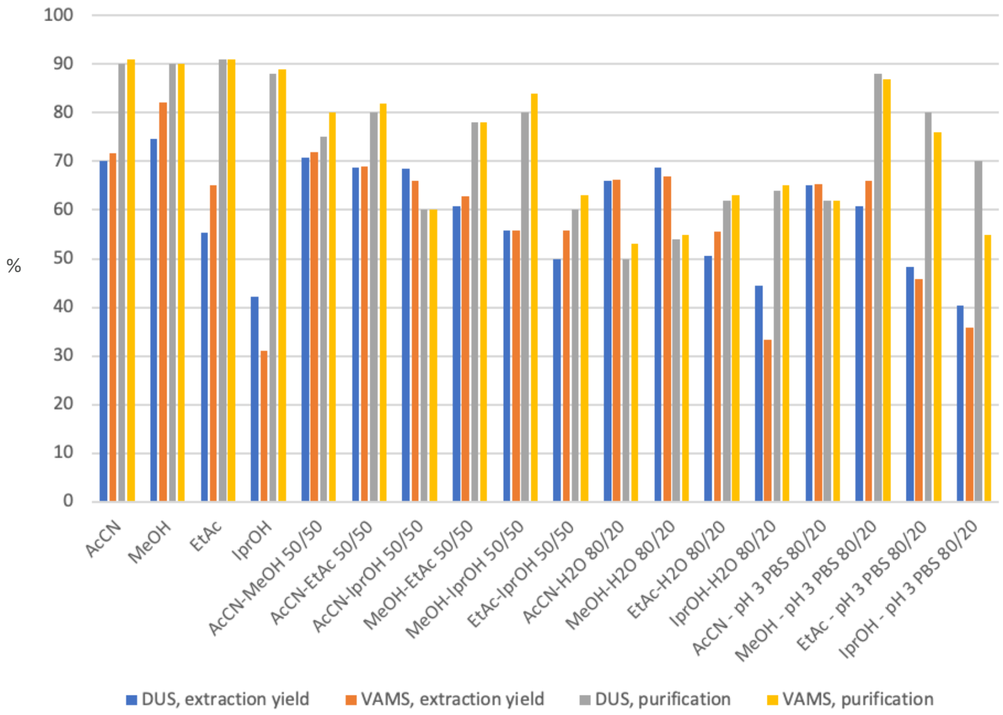

Figure S4. Extraction yield and purification assay results: extraction solvent comparison.

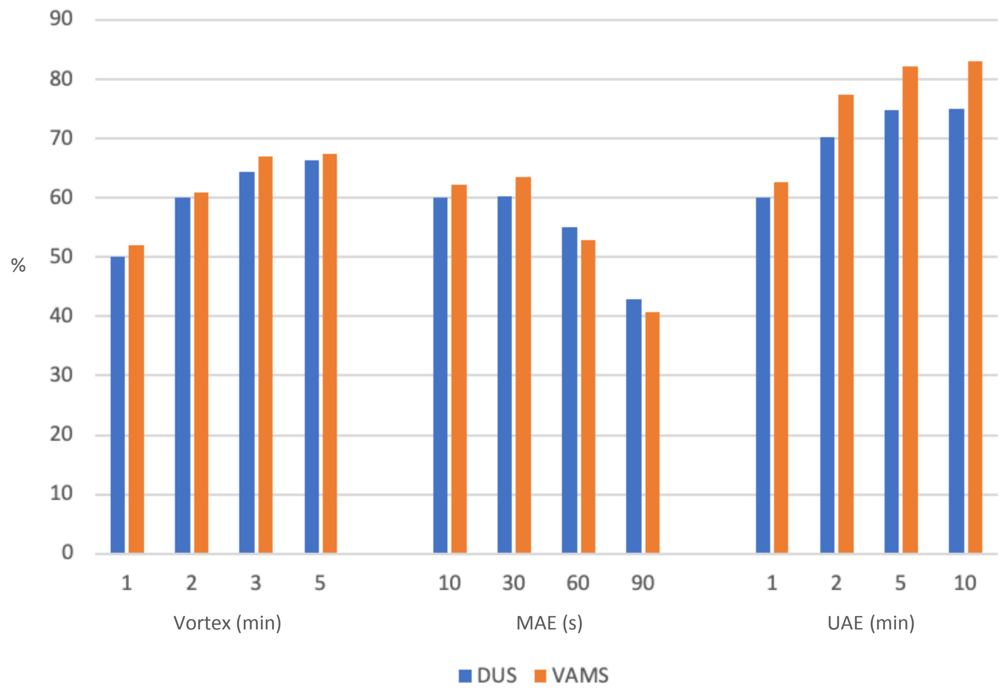

Figure S5. Extraction yield and purification assay results: extraction means comparison. MAE, microwave-assisted extraction; UAE, ultrasound-assisted extraction.

Table S1. Performance comparison of existing methods with the proposed method for anabolic steroid analysis.

Method	Technique	No. of analytes	Sample volume, µL	Sample pretreatment	LOD LOQ, ng/mL	Absolute recovery, %	Matrix Effect, RE %
Protti *et al.* [a]	LC-MS/MS	3 Endog 10 Exog.	30	Solvent extraction	≤0.5 ≤1.5	>76	<12
Wang *et al.*	GC-IRMS	7 Endog.	5000	SPE + Semiprep. HPLC + derivat.	<2 n.d.	n.d.	n.d.
Van Eenoo *et al.*	GC-MS/MS	13 Endog.	1000	LLE + derivat.	≤20 ≤48	n.d.	n.d.
Raro *et al.*	GC-MS/MS	16 Exog.	2500	LLE + derivat.	≤1 n.d.	>47	n.d.
Cha *et al.*	GC-MS/MS LC-MS/MS	79	2000	SPE + derivat. SPE	≤20 n.d.	n.d.	n.d.
Guo *et al.*	LC-MS/MS	1 Endog. 3 Exog.	1000	Online TF-SPE	≤0.005 n.d.	78-150	<22
Saito *et al.*	LC-MS/MS	3 Endog. 5 Exog.	100	In-tube SPME	≤0.182 n.d.	n.d.	n.d.
Jeon *et al.*	LC-MS/MS	44 Exog.	≤ 5000	DSPE + LLE	≤10 n.d.	n.d.	n.d.
Leinonen *et al.*	LC-MS/MS	10 Exog.	2500	LLE	≤2 n.d.	>26	n.d.
Fragkaki *et al.*	LC-MS/MS	34	2500	Derivat. + LLE	≤5 n.d.	n.d.	<2

[a] Proposed method.

Abbreviations: DSPE, dispersive solid phase extraction; GC, gas chromatography; IR, isotopic ratio; LC or HPLC, liquid chromatography; LLE, liquid-liquid extraction; MS, mass spectrometry; n.d., not declared; SPE, solid phase extraction; SPME, solid phase microextraction; TF, turbulent flow.

Table S2. Passing-Bablok regression parameters.

Comparison	n	Concentration range, ng/mL	Slope [95% CI]	Intercept [95% CI]	r^2
VAMS - urine	18	0-150	1.0079 [0.9950-1.0148]	-0.0146 [-02604-0.7920]	0.9996
	11	150-1000	0.9997 [0.8979-1.0086]	-1.8172 [-5.5416-4.5305]	0.9999
DUS - urine	18	0-150	1.0131 [0.9782-1.0485]	0.1656 [-2.0051-0.7091]	0.9996
	11	150-1000	0.9942 [0.9741-1.0306]	-1.3744 [-16.2586-5.6053]	0.9992
